# Transcriptional Modulation of Polyamine Metabolism in Fruit Species Under Abiotic and Biotic Stress

**DOI:** 10.3389/fpls.2019.00816

**Published:** 2019-07-02

**Authors:** Ana Margarida Fortes, Patricia Agudelo-Romero, Diana Pimentel, Noam Alkan

**Affiliations:** ^1^ Faculdade de Ciências de Lisboa, Department of Plant Biology, Biosystems and Integrative Sciences Institute, Universidade de Lisboa, Lisbon, Portugal; ^2^ School of Molecular Science, The University of Western Australia, Perth, WA, Australia; ^3^ ARC Centre of Excellence in Plant Energy Biology, The University of Western Australia, Perth, WA, Australia; ^4^ Telethon Kids Institute, University of Western Australia, Nedlands, WA, Australia; ^5^ Department of Postharvest Science of Fresh Produce, Agricultural Research Organization, Volcani Center, Rishon LeZion, Israel

**Keywords:** abiotic stress, biotic stress, fruit ripening, grape, polyamine, tomato

## Abstract

Polyamines are growth regulators that have been widely implicated in abiotic and biotic stresses. They are also associated with fruit set, ripening, and regulation of fruit quality-related traits. Modulation of their content confers fruit resilience, with polyamine application generally inhibiting postharvest decay. Changes in the content of free and conjugated polyamines in response to stress are highly dependent on the type of abiotic stress applied or the lifestyle of the pathogen. Recent studies suggest that exogenous application of polyamines or modulation of polyamine content by gene editing can confer tolerance to multiple abiotic and biotic stresses simultaneously. In this review, we explore data on polyamine synthesis and catabolism in fruit related to pre- and postharvest stresses. Studies of mutant plants, priming of stress responses, and treatments with polyamines and polyamine inhibitors indicate that these growth regulators can be manipulated to increase fruit productivity with reduced use of pesticides and therefore, under more sustainable conditions.

## Introduction

Polyamines (PAs) are small aliphatic amines that regulate various cellular functions. These compounds present at least two amino groups; the diamine putrescine (Put), the triamine spermidine (Spd), and the tetraamine spermine (Spm) are the most common PAs in plants (Mattoo et al., 2010). PAs often occur as free molecular bases, but they can also be covalently associated with small molecules, namely, phenolic acids (conjugated forms), and with various macromolecules such as proteins (bound forms) (Mattoo et al., 2010).

PAs are growth regulators that have been implicated in abiotic and biotic stresses (Cona et al., 2006; Cuevas et al., 2008; Marina et al., 2008; Alcázar et al., 2010; Gonzalez et al., 2011; Nambeesan et al., 2012; Agudelo-Romero et al., 2014; Minocha et al., 2014; Pál et al., 2015), as well as in plant morphogenesis and development (Applewhite et al., 2000; Fortes et al., 2010, 2011; Tiburcio et al., 2014; Jancewicz et al., 2016), senescence (Pandey et al., 2000; Sobieszczuk-Nowicka, 2017), and fruit development and ripening (Mattoo and Handa, 2008; Agudelo-Romero et al., 2013, 2014; Tavladoraki et al., 2016). Several publications have suggested that the role played by these growth regulators in plant–microbe interactions is either exerted directly by PAs functioning as signaling molecules or mediated through the products of their catabolism together with jasmonic acid (JA), abscisic acid (ABA), salicylic acid (SA), auxins, cytokinins, and ethylene (Jiménez-Bremont et al., 2014).

In plants, Put is synthesized through arginine decarboxylase (ADC) and/or ornithine decarboxylase (ODC) (Alcázar et al., 2010). Arginase hydrolyzes arginine to urea and ornithine. Conversion of Put to Spd and Spm requires the activity of Spd synthase and Spm synthase, respectively. Thermospermine synthase is involved in the synthesis of the tetraamine thermospermine (Jiménez-Bremont et al., 2014). *S*-adenosylmethionine decarboxylase (SAMDC) carries out a rate-limiting step in the biosynthesis of decarboxylated SAM, which donates the aminopropyl moiety for the biosynthesis of these PAs. Catabolism of PAs involves diamine oxidases (CuAOs) and polyamine oxidases (PAOs). Intracellular levels of PAs are mostly regulated by anabolic and catabolic processes, as well as by their transport and conjugation with phenolic compounds, mainly hydroxycinnamic acids (Jiménez-Bremont et al., 2014; Fortes and Agudelo-Romero, 2018). The transport of PAs into different cell compartments is a crucial step in regulating the intracellular levels of these free forms, thereby interfering with cellular processes. However, only a few PA transporters have been characterized (Jiménez-Bremont et al., 2014). In addition, PAs have been connected to metabolic pathways involving ethylene, γ-aminobutyric acid (GABA), nitric oxide, the Krebs cycle, and ABA (Alcázar et al., 2010).

In this mini-review, we will examine recent data focusing on the modulation of PA metabolism in plants and fruit under pre- and postharvest abiotic stresses and during interactions with pathogens.

## Reprogramming of Polyamine Synthesis, Catabolism, and Conjugation is Involved in Abiotic and Biotic Stress Responses

### Transcriptional Modulation of Polyamine Metabolism Under Individual Stresses

**Figure 1:**
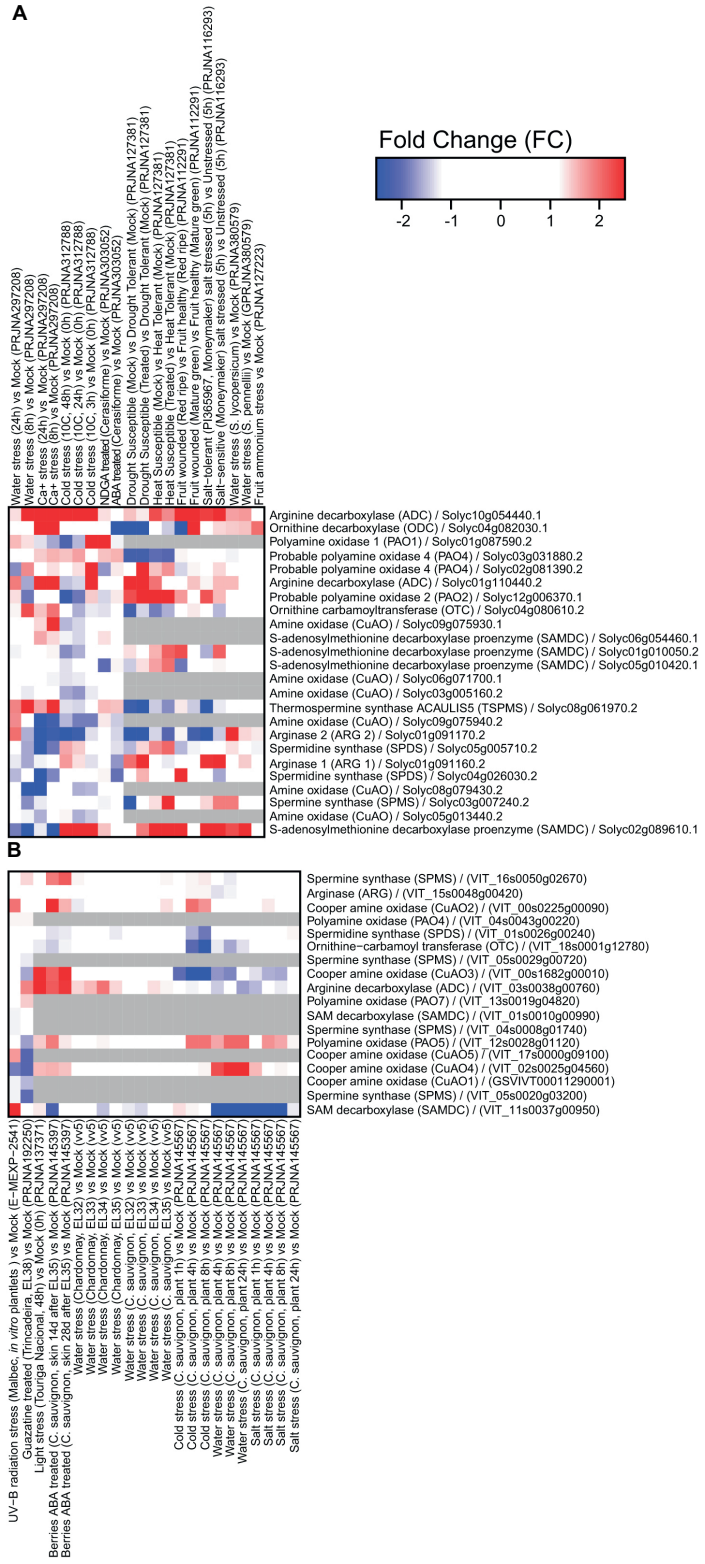
Expression of genes involved in polyamine metabolism in tomato **(A)** and grapevine **(B)** under abiotic stresses: water stress, Ca^+^ treatment, cold, nordihydroguaiaretic acid (NDGA; an ABA biosynthesis blocker) treatment, ABA, drought, heat, wounding, salt treatment, ammonium treatment, UV-B irradiation, guazatine treatment, and light. Tomato heatmaps were generated using RNAseq (SL2.50 genome) and microarray (GPL4741) approaches. Grape heatmaps were generated using two microarray platforms: GrapeGen (GPL11004) and GeneChip (GPL1320). RNAseq data were downloaded from the Sequence Read Archive repository (SRA; https://www.ncbi.nlm.nih.gov/sra) and microarray data were downloaded from the Gene Expression Omnibus repository (GEO; https://www.ncbi.nlm.nih.gov/geo/) using the GEOquery R library.

**Figure 2:**
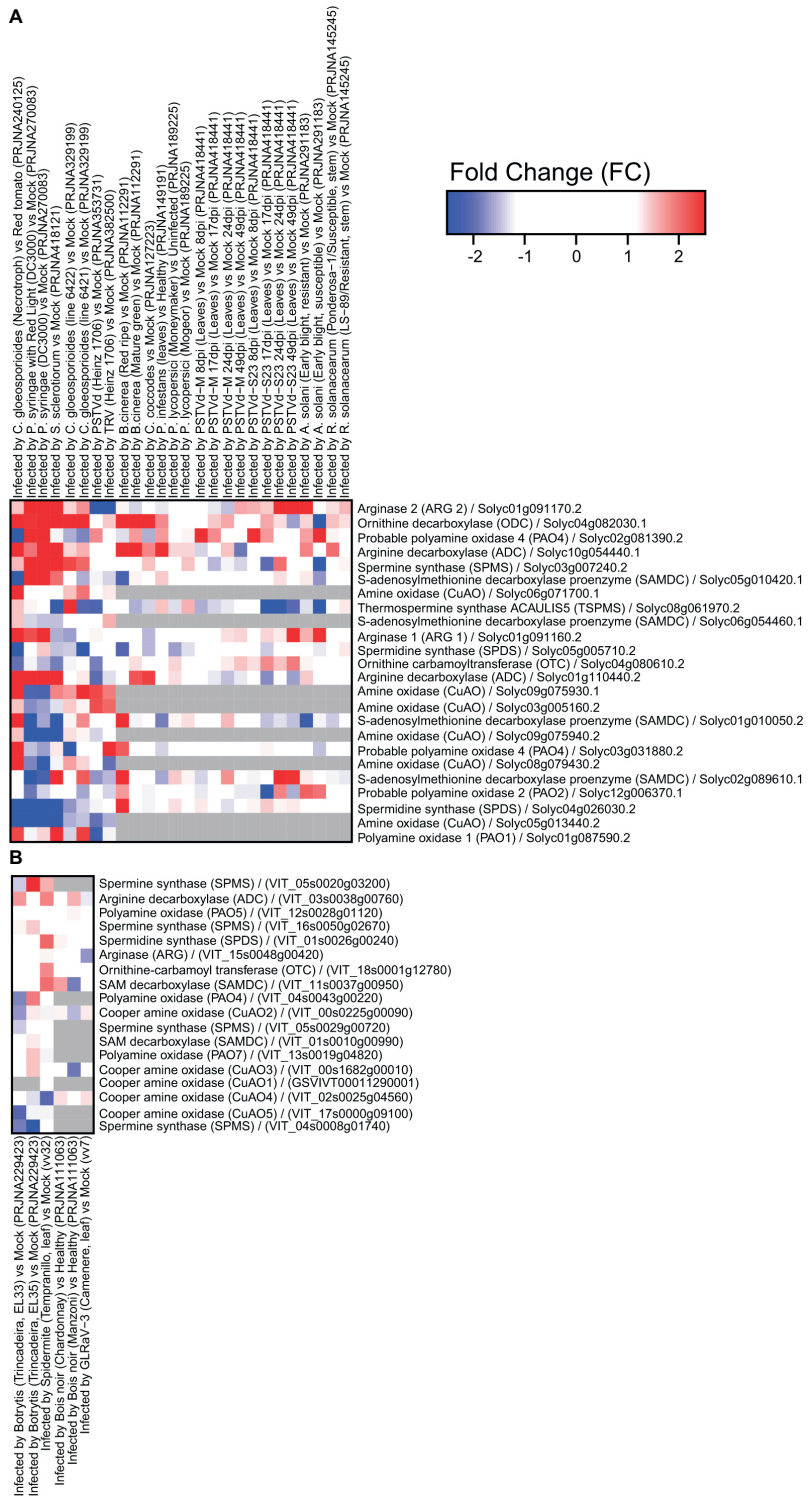
Expression of genes involved in polyamine metabolism in tomato **(A)** and grapevine **(B)** under biotic stresses: *Colletotrichum gloeosporioides, Pseudomonas syringae, Sclerotinia sclerotiorum*, potato spindle tuber viroid (PSTVd), tobacco rattle virus (TRV), *Botrytis cinerea, Colletotrichum coccodes, Phytophthora infestans, Pyrenochaeta lycopersici, Alternaria solani, Ralstonia solanacearum*, spider mite, Bois noir, and grapevine leafroll-associated virus 3 (GLRaV-3). Tomato heatmaps were generated using RNAseq (SL2.50 genome) and microarray (GPL4741) approaches. Grape heatmaps were generated using three microarray platforms: NimbleGen (GPL17894), GrapeGen (GPL11004) and GeneChip (GPL1320). RNAseq data were downloaded from the Sequence Read Archive repository (SRA; https://www.ncbi.nlm.nih.gov/sra) and microarray data were downloaded from the Gene Expression Omnibus repository (GEO; https://www.ncbi.nlm.nih.gov/geo/) using the GEOquery R library.

Several datasets have been obtained in tomato and grape related to transcriptome reprogramming under abiotic and biotic stresses (Figures 1, 2). It is clear that extensive modulation of PA metabolism occurs in both leaves and fruit in response to drought, salt, heat, and a variety of pathogens, such as viruses, fungi, and bacteria. Responses to abiotic stress involve mainly upregulation of genes encoding enzymes involved in PA biosynthesis, namely, ADC and SAMDC. However, *SAMDC* was found to be downregulated in grapevine under water and salt stresses. In this respect, it is interesting to note that different functional roles have been observed with the genetic divergence of the *Arabidopsis thaliana SAMDC* gene family (Majumdar et al., 2017). The gene encoding thermospermine synthase was upregulated in tomato under water stress and upon calcium treatment. In tomato, *ODC* was upregulated in response to calcium and downregulated in ABA-treated plants and in those exposed to water stress. This gene seemed to be more modulated upon biotic stress. The gene encoding arginase was downregulated in response to several abiotic stresses in tomato, suggesting that synthesis of PAs occurs preferentially through ADC. Interestingly, transcriptional modulation of genes involved in PA catabolism is strongly dependent on the type of abiotic stress applied and likely involves activity reprogramming of different isoenzymes, as suggested by the differential expression of several genes.

On the other hand, changes in PA metabolism in response to biotic stress seem to involve mainly inhibition of PA catabolism *via* downregulation of genes encoding CuAO and PAO. The gene encoding Spd synthase was downregulated in tomato infected with the fungus *Pseudomonas syringae*, and a virus. In addition, genes involved in PA biosynthesis (encoding arginase, ODC, and ADC) were upregulated (Figure 2). These results suggest activation of PA metabolism in response to biotic stresses such as bacteria and fungi, but less pronounced involvement in response to viruses.

### Polyamine Metabolism Under Individual and Multiple Stresses Using Transgenic, Biochemical, and Physiological Approaches

Transgenic tomato plants overexpressing the human *SAMDC* gene had higher PA content than the wild type. This transgenic tomato line showed increased resistance to two important fungal pathogens, *Fusarium oxysporum* causing Fusarium wilt and *Alternaria solani* causing early blight, as well as tolerance to multiple abiotic stresses, such as salinity, drought, cold, and high temperatures (Hazarika and Rajam, 2011). Interestingly, these transgenic plants also showed higher conversion of free Put and Spd to conjugated PAs when infected with pathogens (Hazarika and Rajam, 2011). In fact, conjugated PAs have been shown to have antimicrobial properties (Walters, 2003). These PAs contribute to cell-wall strengthening, thereby protecting it against the activity of microbial hydrolytic enzymes [reviewed by Fortes and Agudelo-Romero (2018)].

Similarly, transgenic eggplants overexpressing the oat *ADC* gene acquired resistance to Fusarium wilt disease (Prabhavathi and Rajam, 2007). These plants showed increased ADC activity and accumulation of PAs, particularly the conjugated forms of Put and Spm. Since CuAO activity was also enhanced, it was suggested that the acquisition of resistance might involve both PA biosynthesis and degradation.

Other studies that did not involve transgenic approaches also revealed the importance of PAs in resistance to biotic stress. One study demonstrated that NH_4_^+^ induces resistance to *P. syringae* pv*. tomato DC3000 (Pst)*, by causing mild toxicity in tomato plants and inducing basal H_2_O_2_, ABA, and Put accumulation (Fernández-Crespo et al., 2015). Treatment with inhibitors of Put accumulation showed that Put plays a role in resistance to *Pst* in tomato plants (Fernández-Crespo et al., 2015). On the other hand, the increase in H_2_O_2,_ leading to a strong and rapid oxidative burst, was partially attributed to the activity of CuAOs. Export of PAs to the apoplast is a common source of H_2_O_2_ in abiotic stress and in host- and nonhost hypersensitive responses during pathogen infection (Yoda et al., 2009; Pottosin et al., 2014). Oxidation of PAs can generate an oxidative burst, leading to induction of defense-response genes and the hypersensitive response (Cona et al., 2006; Yoda et al., 2009). The activation of PA metabolism by NH_4_^+^ supplementation was suggested to be mediated by ABA-dependent signaling pathways (Toumi et al., 2010). Similarly, NH_4_^+^ application and *Colletotrichum coccodes* inoculation of tomato fruit induced NADPH oxidase, leading to an oxidative burst, activation of the SA pathway, and upregulation of *ODC* and other PA-related genes, ultimately resulting in programmed cell death (Alkan et al., 2009, 2012).

Under conditions of abiotic and biotic stress, PA oxidation might alter cell redox homeostasis and modulate hormone signaling (Moschou et al., 2008; Pál et al., 2015; Seifi and Shelp, 2019). However, PA synthesis and oxidation control its homeostasis and can lead to PA excess or deficiency, which could lead to susceptibility to stress (Aziz et al., 1999; Nambeesan et al., 2012; Hatmi et al., 2015). Thus, fine-tuning of PAs could be important to coordinating stress responses (Pál et al., 2015).

Interestingly, drought stress was also described to prime the immune response of grapevine against *Botrytis cinerea* infection *via* modulation of PA biosynthesis and catabolism. Drought stress led to upregulation of the genes encoding ADC, CuAOs, and PAOs and their corresponding enzymes’ activities (Hatmi et al., 2015). Plants from a *B. cinerea*-tolerant cultivar subjected to drought stress exhibited significantly higher Put accumulation and a decrease in Spd and Spm levels as compared to plants from the sensitive cultivar. Hatmi et al. (2015) indicated that PA synthesis and oxidation and increased contents of some PA-related amino acids, together with increased content of stilbenes and upregulation of immune response-related genes were involved in the increased tolerance to *B. cinerea*. CuAO and PAO inhibitors attenuated drought-induced defense responses and enhanced disease susceptibility in grapevine. Furthermore, grapes treated with guazatine, a potent inhibitor of PAO activity, showed downregulation of genes encoding CuAOs (Figure 1) and the pathogenesis-related protein 1 precursor (*PR1* gene) involved in the biotic stress response (Agudelo-Romero et al., 2014).

Upon infection with *B. cinerea*, grape fruit from a highly susceptible cultivar presented upregulation of *CuAO* and *ODC* at an early stage of ripening together with increased transcription of stilbene synthases involved in stilbene synthesis (Agudelo-Romero et al., 2015). However, with the onset of ripening, these genes were no longer upregulated in infected berries but others involved in PA metabolism such as *Spm synthase* (Figure 2). Similarly, accumulation of Spd in transgenic tomato (overexpressing the yeast Spd synthase gene) was associated with weakened ethylene-induced defense responses, thereby increasing the fruit’s susceptibility to *B. cinerea* (Nambeesan et al., 2012). Osmotic stress also induced PA accumulation and inhibited the defense response in ripe berries after *B. cinerea* infection (Hatmi et al., 2018). In fact, the plant’s response to individual stresses may differ from that to multiple stresses, which could lead to opposite effects on PA metabolism. However, grapevine plants exposed to osmotic stress that induced PA oxidation and later inoculated with *B. cinerea* showed a great reduction in CuAO and PAO activities, consistent with the enhanced levels of PAs and impaired defense responses to the fungus (Hatmi et al., 2014).

It is not known whether fungi can reprogram PA metabolism in fruit, or how this may differ depending on genotype, ripening stage and fruit susceptibility. However, interactions between microbial effectors and plant enzymes involved in PA metabolism have been reported [reviewed by Jiménez-Bremont et al. (2014)]. Microbes can also produce PAs and alter plant physiology and resilience to stress (Kim et al., 2013; Jiménez-Bremont et al., 2014). In one study, the effector AvrBsT of *Xanthomonas campestris* pv. *vesicatoria* (Xcv) interacted with ADC, leading to induction of a hypersensitive response in bell pepper fruit (Kim et al., 2013). In bell pepper, the gene *CaADC1* is constitutively expressed in stems, roots, flowers, and fruit, but not leaves. However, *CaADC1* was highly induced in leaves during avirulent (incompatible) Xcv infection compared to the mock control or virulent (compatible) Xcv infection. Silencing of *CaADC1* in bell pepper leaves significantly compromised nitric oxide and H_2_O_2_ accumulation as well as cell-death induction, leading to enhanced avirulent Xcv growth during infection (Kim et al., 2013). Based on these findings, the authors suggested that *CaADC1* acts as a key defense and cell-death regulator *via* mediation of PA metabolism.

## Exogenous Application of Polyamines Affects Fruit Resistance to Abiotic and Biotic Stresses

Chilling injury results in a significant increase in PA levels in many fruit (Serrano et al., 1997, 1998; González-Aguilar et al., 2000; Rodriguez et al., 2001), as does mechanical damage (Valero et al., 1998; Martínez-Romero et al., 1999, 2000; Pérez-Vicente et al., 2002). This suggests that PAs protect fruit from abiotic stresses, due to their ability to maintain membrane integrity and possibly activate the JA-related defense pathway (Radhakrishnan and Lee, 2013; Tanou et al., 2014). Indeed, postharvest application of PAs (1 mM Put or Spd) alleviated chilling injury of apricot during storage at 1°C (Koushesh saba et al., 2012). Similarly, postharvest application of 1–2 mM Put or 1 mM Spd reduced chilling injury of pomegranate stored at 2°C (Mirdehghan et al., 2007; Barman et al., 2011).

During growth and ripening of both climacteric and nonclimacteric fruit, the natural level of PAs changes (Fortes and Agudelo-Romero, 2018): during the early phase of fruit growth and cell division, PA levels are high; during fruit ripening and senescence, PA levels usually decline, with a few exceptions [Liu et al., 2006; reviewed by Valero et al. (2002)]. During fruit ripening and senescence, there is crosstalk between ethylene and PAs (Pandey et al., 2000; Valero et al., 2002) as SAM is a common precursor for both growth regulators. Therefore, ethylene and PAs may induce or delay fruit ripening and senescence in opposite manners. Indeed, postharvest application of PAs to fruit led to inhibition of ethylene emission. However, the percentage of inhibition was dependent on the ethylene climacteric peak: as the fruit emitted less ethylene, higher inhibition was observed [reviewed by Valero et al. (2002)]. Thus, one of the main effects of PA application is inhibition of fruit ripening, affecting color change, decreasing fruit softening, and delaying ethylene emission and respiration (Valero et al., 2002).

Polyamines may also delay fruit softening by attaching to pectin elements in the cell wall, resulting in increased fruit firmness. This binding blocks cell wall-degrading enzymes’ access to the cell-wall matrix (Valero et al., 1999). On the other hand, a delay in fruit softening and ripening is strongly correlated with increased fruit resistance to fungal pathogens and reduced postharvest decay (Cantu et al., 2008). Polyamines also function as signaling molecules that interact with JA, ABA, and SA (Jiménez-Bremont et al., 2014)—hormones that activate broad defense responses against pathogens in fruit (Alkan and Fortes, 2015).

In accordance with the PAs’ effects on fruit ripening, softening, and defense hormones, a number of studies have shown that in most cases, postharvest application of PAs inhibits fruit ripening and softening while reducing postharvest decay. One of the most significant effects of PA infiltration after harvest was its contribution to fruit firmness in apple (Kramer et al., 1991), strawberry (Ponappa et al., 1993), apricot, peach (Martínez-Romero et al., 2002; Valero et al., 2002), and lemon (Martínez-Romero et al., 1999). Preharvest treatments were similarly effective at increasing fruit firmness (Bregoli et al., 2002).

Postharvest application of about 0.5–1 mM PA inhibited the ripening of plum, kiwi and mango fruit, as reflected by reduced ethylene emission, respiration, and inhibition of softening, thereby prolonging shelf life (Serrano et al., 2003; Petkou et al., 2004; Malik and Zora, 2005). Similarly, preharvest application of PAs inhibited fruit ripening and the expression of genes involved in ethylene synthesis in nectarine and plum (Torrigiani et al., 2004; Khan et al., 2007). In plum, preharvest treatments inhibited ripening and softening better than postharvest treatments. In addition, an increased concentration of 0.3–2 mM Put applied postharvest inhibited strawberry ripening (firmness, total soluble solids, *and* titratable acidity) and was correlated to a delay in rotting caused by fungal pathogens (Khosroshahi et al., 2007). Postharvest application of 100 mg/L Put, Spd, or Spm inhibited decay accumulation in mandarin as well (Zheng and Zhang, 2004). Similarly, overexpression of the yeast Spd synthase gene in tomato led to the accumulation of PAs, reduced ripening and softening, and consequently, reduced decay symptoms (Nambeesan et al., 2010). However, a high concentration of PAs could result in fruit injuries, such as black spot in apples (Kramer et al., 1991), and in the induction of postharvest decay caused by *B. cinerea* in grapes (Nambeesan et al., 2012).

## Conclusions and Perspectives

Many of the studies conducted to date have indicated that PA synthesis, conjugation, and catabolism play important roles in abiotic and biotic stress responses in fruit. However, the analysis of PA-metabolism reprogramming by biotic stress is complicated by the fact that both plants and microbes can synthetize PAs. Changes in gene expression might be due to plant defense responses induced against the pathogen, or they might be triggered by the pathogen’s virulence mechanisms which may differ according to the pathogen’s lifestyle. Biotrophs might benefit from reduced PA oxidation, whereas necrotrophs exploit the generation of an oxidative burst (due to increased PA catabolism) for their pathogenicity (Jiménez-Bremont et al., 2014). What is clear, however, is that the accumulation of free and conjugated PAs—under tight regulation by mechanisms controlling PA biosynthesis and catabolism—plays an important role in host–pathogen interactions, which involve the oxidative stress response, strengthening of the fruit cell wall, and modulation of ABA- and ethylene-related pathways.

Moreover, the stress response might differ with plant species and their metabolism, tissue-specific gene expression, and the interactions among other growth regulators and defense-signaling pathways. The molecular mechanisms regulating these processes need to be elucidated through the use of transgenic and mutant plant lines. However, this is a challenge for herbaceous and woody fruit species, for which transgenesis protocols have not yet been established. Studies in these plants have been mostly performed by testing treatments with PAs and inhibitors of PA metabolism.

Some PAs that were scarcely investigated in the past are receiving more attention today, namely, thermospermine and caldopentamine (Jiménez-Bremont et al., 2014), and products of PA catabolism such as GABA and 1,3 diaminopropane. Studying these may reveal new mechanisms involved in tolerance to abiotic and biotic stresses. Another level of complexity is added when one considers the role of PAs in epigenetic regulation, a topic that has been little explored. PAs can interact with chromatin, eventually leading to epigenetic modifications of DNA and histones [reviewed by Jiménez-Bremont et al. (2014)]; this opens new and exciting frontiers for research focusing on how PA metabolism affects fruit resilience. In addition, the study of PA metabolism in fruit ripening has highlighted the possible application of these natural polycations for the control of ripening and postharvest decay.

Many challenges still remain in PA research toward increasing plants’ tolerance to stresses, in particular in fruit species. Manipulation of PA levels by either modulating their biosynthesis or catabolism or eventually conjugating them with other compounds may contribute in the future to obtaining better yields under more sustainable conditions with reduced application of fungicides.

## Author Contributions

AF designed the mini-review. AF and NA wrote the manuscript with some inputs from DP. PA-R generated the heatmaps.

### Conflict of Interest Statement

The authors declare that the research was conducted in the absence of any commercial or financial relationships that could be construed as a potential conflict of interest.

## References

[ref1] Agudelo-Romero P. Ali K. Choi Y. H. Sousa L. Verpoorte R. Tiburcio A. F.. (2014). Perturbation of polyamine catabolism affects grape ripening of *Vitis vinifera* cv. Trincadeira. Plant Physiol. Biochem. 74, 141–155. 10.1016/j.plaphy.2013.11.002, PMID: 24296250

[ref2] Agudelo-Romero P. Bortolloti C. Pais M. S. Tiburcio A. F. Fortes A. M. (2013). Study of polyamines during grape ripening indicate an important role of polyamine catabolism. Plant Physiol. Biochem. 67, 105–119. 10.1016/j.plaphy.2013.02.024, PMID: 23562795

[ref3] Agudelo-Romero P. Erban A. Rego C. Carbonell-Bejerano P. Nascimento T. Sousa L.. (2015). Transcriptome and metabolome reprogramming in *Vitis vinifera* cv. Trincadeira berries upon infection with *Botrytis cinerea*. J. Exp. Bot. 66, 1769–1785. 10.1093/jxb/eru517, PMID: 25675955 PMC4669548

[ref4] Alcázar R. Altabella T. Marco F. Bortolotti C. Reymond M. Koncz C.. (2010). Polyamines: molecules with regulatory functions in plant abiotic stress tolerance. Planta 231, 1237–1249. 10.1007/s00425-010-1130-0, PMID: 20221631

[ref5] Alkan N. Davydov O. Sagi M. Fluhr R. Prusky D. (2009). Ammonium secretion by *Colletotrichum coccodes* activates host NADPH oxidase activity enhancing host cell death and fungal virulence in tomato fruits. MPMI 22, 1484–1491. 10.1094/MPMI-22-12-1484, PMID: 19888814

[ref6] Alkan N. Fluhr R. Prusky D. (2012). Ammonium secretion during *Colletotrichum coccodes* infection modulates salicylic and jasmonic acid pathways of ripe and unripe tomato fruit. MPMI 25, 85–96. 10.1094/MPMI-01-11-0020, PMID: 22150075

[ref7] Alkan N. Fortes A. M. (2015). Insights into molecular and metabolic events associated with fruit response to post-harvest fungal pathogens. Front. Plant Sci. 6:889. 10.3389/fpls.2015.00889, PMID: 26539204 PMC4612155

[ref8] Applewhite P. B. Kaur-Sawhney R. Galston A. W. (2000). A role for spermidine in the bolting and flowering of Arabidopsis. Physiol. Plant. 108, 314–320. 10.1034/j.1399-3054.2000.108003314.x

[ref9] Aziz A. Martin-Tanguy J. Larher F. (1999). Salt stress-induced proline accumulation and changes in tyramine and polyamine levels are linked to ionic adjustment in tomato leaf discs. Plant Sci. 145, 83–91. 10.1016/S0168-9452(99)00071-0

[ref10] Barman K. Asrey R. Pal R. K. (2011). Putrescine and carnauba wax pretreatments alleviate chilling injury, enhance shelf life and preserve pomegranate fruit quality during cold storage. Sci. Hortic. 130, 795–800. 10.1016/j.scienta.2011.09.005

[ref11] Bregoli A. M. Scaramagli S. Costa G. Sabatini E. Ziosi V. Biondi S.. (2002). Peach (*Prunus persica*) fruit ripening: aminoethoxyvinylglycine (AVG) and exogenous polyamines affect ethylene emission and flesh firmness. Physiol. Plant. 114, 472–481. 10.1034/j.1399-3054.2002.1140317.x, PMID: 12060270

[ref12] Cantu D. Vicente A. R. Labavitch J. M. Bennett A. B. Powell A. L. T. (2008). Strangers in the matrix: plant cell walls and pathogen susceptibility. Trends Plant Sci. 13, 610–617. 10.1016/j.tplants.2008.09.002, PMID: 18824396

[ref13] Cona A. Rea G. Angelini R. Federico R. Tavladoraki P. (2006). Functions of amine oxidases in plant development and defence. Trends Plant Sci. 11, 80–88. 10.1016/j.tplants.2005.12.009, PMID: 16406305

[ref14] Cuevas J. C. López-Cobollo R. Alcázar R. Zarza X. Koncz C. Altabella T.. (2008). Putrescine is involved in Arabidopsis freezing tolerance and cold acclimation by regulating abscisic acid levels in response to low temperature. Plant Physiol. 148, 1094–1105. 10.1104/pp.108.122945, PMID: 18701673 PMC2556839

[ref15] Fernández-Crespo E. Scalschi L. Llorens E. García-Agustín P. Camañes G. (2015). NH4+ protects tomato plants against *Pseudomonas syringae* by activation of systemic acquired acclimation. J. Exp. Bot. 66, 6777–6790. 10.1093/jxb/erv382, PMID: 26246613 PMC4623687

[ref16] Fortes A. M. Agudelo-Romero P. (2018). “Polyamine metabolism in climacteric and non-climacteric fruit ripening” in Polyamines. eds. Alcázar R. Tiburcio A. F. (New York, NY: Springer), 433–447. 10.1007/978-1-4939-7398-9_36 29080186

[ref17] Fortes A. M. Agudelo-Romero P. Silva M. S. Ali K. Sousa L. Maltese F.. (2011). Transcript and metabolite analysis in Trincadeira cultivar reveals novel information regarding the dynamics of grape ripening. BMC Plant Biol. 11:149. 10.1186/1471-2229-11-149, PMID: 22047180 PMC3215662

[ref18] Fortes A. M. Santos F. Pais M. S. (2010). Organogenic nodule formation in hop: a tool to study morphogenesis in plants with biotechnological and medicinal applications. Biomed. Res. Int. 2010:583691. 10.1155/2010/583691 PMC2929504 20811599

[ref19] Gonzalez M. E. Marco F. Minguet E. G. Carrasco-Sorli P. Blázquez M. A. Carbonell J.. (2011). Perturbation of spermine synthase gene expression and transcript profiling provide new insights on the role of the tetraamine spermine in Arabidopsis defense against *Pseudomonas viridiflava*. Plant Physiol. 156, 2266–2277. 10.1104/pp.110.171413, PMID: 21628628 PMC3149955

[ref20] González-Aguilar G. A. Gayosso L. Cruz R. Fortiz J. Báez R. Wang C. Y. (2000). Polyamines induced by hot water treatments reduce chilling injury and decay in pepper fruit. Postharvest Biol. Technol. 18, 19–26. 10.1016/S0925-5214(99)00054-X

[ref21] Hatmi S. Gruau C. Trotel-Aziz P. Villaume S. Rabenoelina F. Baillieul F.. (2015). Drought stress tolerance in grapevine involves activation of polyamine oxidation contributing to improved immune response and low susceptibility to *Botrytis cinerea*. J. Exp. Bot. 66, 775–787. 10.1093/jxb/eru436, PMID: 25385768

[ref22] Hatmi S. Trotel-Aziz P. Villaume S. Couderchet M. Clément C. Aziz A. (2014). Osmotic stress-induced polyamine oxidation mediates defence responses and reduces stress-enhanced grapevine susceptibility to *Botrytis cinerea*. J. Exp. Bot. 65, 75–88. 10.1093/jxb/ert351, PMID: 24170740

[ref23] Hatmi S. Villaume S. Trotel-Aziz P. Barka E. A. Clément C. Aziz A. (2018). Osmotic stress and ABA affect immune response and susceptibility of grapevine berries to gray mold by priming polyamine accumulation. Front. Plant Sci. 9:1010. 10.3389/fpls.2018.01010, PMID: 30050554 PMC6050403

[ref24] Hazarika P. Rajam M. V. (2011). Biotic and abiotic stress tolerance in transgenic tomatoes by constitutive expression of S-adenosylmethionine decarboxylase gene. Physiol. Mol. Biol. Plants 17, 115–128. 10.1007/s12298-011-0053-y, PMID: 23573001 PMC3550545

[ref25] Jancewicz A. L. Gibbs N. M. Masson P. H. (2016). Cadaverine’s functional role in plant development and environmental response. Front. Plant Sci. 7:870. 10.3389/fpls.2016.00870, PMID: 27446107 PMC4914950

[ref26] Jiménez-Bremont J. F. Marina M. Guerrero-González M. d. l. L. Rossi F. R. Sánchez-Rangel D. Rodríguez-Kessler M.. (2014). Physiological and molecular implications of plant polyamine metabolism during biotic interactions. Front. Plant Sci. 5:95. 10.3389/fpls.2014.00095, PMID: 24672533 PMC3957736

[ref27] Khan A. S. Singh Z. Abbasi N. A. (2007). Pre-storage putrescine application suppresses ethylene biosynthesis and retards fruit softening during low temperature storage in ‘Angelino’ plum. Postharvest Biol. Technol. 46, 36–46. 10.1016/j.postharvbio.2007.03.018

[ref28] Khosroshahi M. R. Z. Esna-Ashari M. Ershadi A. (2007). Effect of exogenous putrescine on post-harvest life of strawberry (*Fragaria ananassa* Duch.) fruit, cultivar Selva. Sci. Hortic. 114, 27–32. 10.1016/j.scienta.2007.05.006

[ref29] Kim N. H. Kim B. S. Hwang B. K. (2013). Pepper arginine decarboxylase is required for polyamine and γ-aminobutyric acid signaling in cell death and defense response. Plant Physiol. 162, 2067–2083. 10.1104/pp.113.217372, PMID: 23784462 PMC3729783

[ref30] Koushesh saba M. Arzani K. Barzegar M. (2012). Postharvest polyamine application alleviates chilling injury and affects apricot storage ability. J. Agric. Food Chem. 60, 8947–8953. 10.1021/jf302088e, PMID: 22867007

[ref31] Kramer G. F. Wang C. Y. Conway W. S. (1991). Inhibition of softening by polyamine application in ‘Golden Delicious’ and ‘McIntosh’ apples. J. Am. Soc. Hortic. Sci. 116, 813–817.

[ref32] Liu J.-H. Honda C. Moriguchi T. (2006). Involvement of polyamine in floral and fruit development. JARQ 40, 51–58. 10.6090/jarq.40.51

[ref33] Majumdar R. Shao L. Turlapati S. A. Minocha S. C. (2017). Polyamines in the life of Arabidopsis: profiling the expression of S-adenosylmethionine decarboxylase (SAMDC) gene family during its life cycle. BMC Plant Biol. 17:264. 10.1186/s12870-017-1208-y, PMID: 29281982 PMC5745906

[ref34] Malik A. U. Zora S. (2005). Pre-storage application of polyamines improves shelf-life and fruit quality of mango. J. Hortic. Sci. Biotechnol. 80, 363–369. 10.1080/14620316.2005.11511945

[ref35] Marina M. Maiale S. J. Rossi F. R. Romero M. F. Rivas E. I. Gárriz A.. (2008). Apoplastic polyamine oxidation plays different roles in local responses of tobacco to infection by the necrotrophic fungus *Sclerotinia sclerotiorum* and the biotrophic bacterium *Pseudomonas viridiflava*. Plant Physiol. 147, 2164–2178. 10.1104/pp.108.122614, PMID: 18583531 PMC2492638

[ref36] Martínez-Romero D. Serrano M. Carbonell A. Burgos L. Riquelme F. Valero D. (2002). Effects of postharvest putrescine treatment on extending shelf life and reducing mechanical damage in apricot. J. Food Sci. 67, 1706–1712. 10.1111/j.1365-2621.2002.tb08710.x

[ref37] Martínez-Romero D. Valero D. Serrano M. Burló F. Carbonell A. Burgos L. (2000). Exogenous polyamines and gibberellic acid effects on peach (*Prunus persica* L.) storability improvement. J. Food Sci. 65, 288–294. 10.1111/j.1365-2621.2000.tb15995.x

[ref38] Martínez-Romero D. Valero D. Serrano M. Martínez-Sánchez F. Riquelme F. (1999). Effects of post-harvest putrescine and calcium treatments on reducing mechanical damage and polyamines and abscisic acid levels during lemon storage. J. Sci. Food Agric. 79, 1589–1595. 10.1002/(SICI)1097-0010(199909)79:12<1589::AID-JSFA403>3.0.CO;2-J

[ref39] Mattoo A. K. Handa A. K. (2008). Higher polyamines restore and enhance metabolic memory in ripening fruit. Plant Sci. 174, 386–393. 10.1016/j.plantsci.2008.01.011

[ref40] Mattoo A. K. Minocha S. C. Minocha R. Handa A. K. (2010). Polyamines and cellular metabolism in plants: transgenic approaches reveal different responses to diamine putrescine versus higher polyamines spermidine and spermine. Amino Acids 38, 405–413. 10.1007/s00726-009-0399-4, PMID: 19956999

[ref41] Minocha R. Majumdar R. Minocha S. C. (2014). Polyamines and abiotic stress in plants: a complex relationship1. Front. Plant Sci. 5:175. 10.3389/fpls.2014.00175, PMID: 24847338 PMC4017135

[ref42] Mirdehghan S. H. Rahemi M. Castillo S. Martinez-Romero D. Serrano M. Valero D. (2007). Pre-storage application of polyamines by pressure or immersion improves shelf-life of pomegranate stored at chilling temperature by increasing endogenous polyamine levels. Postharvest Biol. Technol. 44, 26–33. 10.1016/j.postharvbio.2006.11.010

[ref43] Moschou P. N. Paschalidis K. A. Roubelakis-Angelakis K. A. (2008). Plant polyamine catabolism: the state of the art. Plant Signal. Behav. 3, 1061–1066. 10.4161/psb.3.12.7172 19513239 PMC2634460

[ref44] Nambeesan S. AbuQamar S. Laluk K. Mattoo A. K. Mickelbart M. V. Ferruzzi M. G.. (2012). Polyamines attenuate ethylene-mediated defense responses to abrogate resistance to *Botrytis cinerea* in tomato. Plant Physiol. 158, 1034–1045. 10.1104/pp.111.188698, PMID: 22128140 PMC3271740

[ref45] Nambeesan S. Datsenka T. Ferruzzi M. G. Malladi A. Mattoo A. K. Handa A. K. (2010). Overexpression of yeast spermidine synthase impacts ripening, senescence and decay symptoms in tomato. Plant J. 63, 836–847. 10.1111/j.1365-313X.2010.04286.x, PMID: 20584149

[ref46] Pál M. Szalai G. Janda T. (2015). Speculation: polyamines are important in abiotic stress signaling. Plant Sci. 237, 16–23. 10.1016/j.plantsci.2015.05.003, PMID: 26089148

[ref47] Pandey S. Ranade S. A. Nagar P. K. Kumar N. (2000). Role of polyamines and ethylene as modulators of plant senescence. J. Biosci. 25, 291–299. 10.1007/BF02703938, PMID: 11022232

[ref48] Pérez-Vicente A. Martínez-Romero D. Carbonell Á. Serrano M. Riquelme F. Guillén F. (2002). Role of polyamines in extending shelf life and the reduction of mechanical damage during plum (*Prunus salicina* Lindl.) storage. Postharvest Biol. Technol. 25, 25–32. 10.1016/S0925-5214(01)00146-6

[ref49] Petkou I. T. Pritsa T. S. Sfakiotakis E. M. (2004). Effects of polyamines on ethylene production, respiration and ripening of kiwifruit. J. Hortic. Sci. Biotechnol. 79, 977–980. 10.1080/14620316.2004.11511876

[ref50] Ponappa T. Scheerens J. C. Miller A. R. (1993). Vacuum infiltration of polyamines increases firmness of strawberry slices under various storage conditions. J. Food Sci. 58, 361–364. 10.1111/j.1365-2621.1993.tb04275.x

[ref51] Pottosin I. Velarde-Buendía A. M. Bose J. Zepeda-Jazo I. Shabala S. Dobrovinskaya O. (2014). Cross-talk between reactive oxygen species and polyamines in regulation of ion transport across the plasma membrane: implications for plant adaptive responses. J. Exp. Bot. 65, 1271–1283. 10.1093/jxb/ert423, PMID: 24465010

[ref52] Prabhavathi V. R. Rajam M. V. (2007). Polyamine accumulation in transgenic eggplant enhances tolerance to multiple abiotic stresses and fungal resistance. Plant Biotechnol. 24, 273–282. 10.5511/plantbiotechnology.24.273

[ref53] Radhakrishnan R. Lee I.-J. (2013). Spermine promotes acclimation to osmotic stress by modifying antioxidant, abscisic acid, and jasmonic acid signals in soybean. J. Plant Growth Regul. 32, 22–30. 10.1007/s00344-012-9274-8

[ref54] Rodriguez S. d. C. López B. Chaves A. R. (2001). Effect of different treatments on the evolution of polyamines during refrigerated storage of eggplants. J. Agric. Food Chem. 49, 4700–4705. 10.1021/jf0001031, PMID: 11600010

[ref55] Seifi H. S. Shelp B. J. (2019). Spermine differentially refines plant defense responses against biotic and abiotic stresses. Front. Plant Sci. 10:117. 10.3389/fpls.2019.00117, PMID: 30800140 PMC6376314

[ref56] Serrano M. Martínez-Madrid M. C. Pretel M. T. Riquelme F. Romojaro F. (1997). Modified atmosphere packaging minimizes increases in putrescine and abscisic acid levels caused by chilling injury in pepper fruit. J. Agric. Food Chem. 45, 1668–1672. 10.1021/jf960866h

[ref57] Serrano M. Martinez-Romero D. Guillén F. Valero D. (2003). Effects of exogenous putrescine on improving shelf life of four plum cultivars. Postharvest Biol. Technol. 30, 259–271. 10.1016/S0925-5214(03)00113-3

[ref58] Serrano M. Pretel M. T. Martínez-Madrid M. C. Romojaro F. Riquelme F. (1998). CO2 treatment of zucchini squash reduces chilling-induced physiological changes. J. Agric. Food Chem. 46, 2465–2468. 10.1021/jf970864c

[ref59] Sobieszczuk-Nowicka E. (2017). Polyamine catabolism adds fuel to leaf senescence. Amino Acids 49, 49–56. 10.1007/s00726-016-2377-y, PMID: 28039518 PMC5241338

[ref60] Tanou G. Ziogas V. Belghazi M. Christou A. Filippou P. Job D.. (2014). Polyamines reprogram oxidative and nitrosative status and the proteome of citrus plants exposed to salinity stress. Plant Cell Environ. 37, 864–885. 10.1111/pce.12204, PMID: 24112028

[ref61] Tavladoraki P. Cona A. Angelini R. (2016). Copper-containing amine oxidases and FAD-dependent polyamine oxidases are key players in plant tissue differentiation and organ development. Front. Plant Sci. 7:824. 10.3389/fpls.2016.00824, PMID: 27446096 PMC4923165

[ref62] Tiburcio A. F. Altabella T. Bitrián M. Alcázar R. (2014). The roles of polyamines during the lifespan of plants: from development to stress. Planta 240, 1–18. 10.1007/s00425-014-2055-9, PMID: 24659098

[ref63] Torrigiani P. Bregoli A. M. Ziosi V. Scaramagli S. Ciriaci T. Rasori A. (2004). Pre-harvest polyamine and aminoethoxyvinylglycine (AVG) applications modulate fruit ripening in stark red gold nectarines (*Prunus persica* L. Batsch). Postharvest Biol. Technol. 33, 293–308. 10.1016/j.postharvbio.2004.03.008

[ref64] Toumi I. Moschou P. N. Paschalidis K. A. Bouamama B. Ben Salem-fnayou A. Ghorbel A. W.. (2010). Abscisic acid signals reorientation of polyamine metabolism to orchestrate stress responses via the polyamine exodus pathway in grapevine. J. Plant Physiol. 167, 519–525. 10.1016/j.jplph.2009.10.022, PMID: 20060616

[ref65] Valero D. Martínez-Romero D. Serrano M. (2002). The role of polyamines in the improvement of the shelf life of fruit. Trends Food Sci. Technol. 13, 228–234. 10.1016/S0924-2244(02)00134-6

[ref66] Valero D. Martínez-Romero D. Serrano M. Riquelme F. (1998). Influence of postharvest treatment with putrescine and calcium on endogenous polyamines, firmness, and abscisic acid in lemon (citrus lemon L. Burm cv. Verna). J. Agric. Food Chem. 46, 2102–2109. 10.1021/jf970866x

[ref67] Valero D. Martínez-Romero D. Serrano M. Riquelme F. (1999). “Polyamine roles on the post-harvest of fruits: a review” in Recent research developments in agricultural and food chemistry. ed. Pandalai S. (Trivandrum, India: Research Signpost), 39–55.

[ref68] Walters D. (2003). Resistance to plant pathogens: possible roles for free polyamines and polyamine catabolism. New Phytol. 159, 109–115. 10.1046/j.1469-8137.2003.00802.x 33873679

[ref69] Yoda H. Fujimura K. Takahashi H. Munemura I. Uchimiya H. Sano H. (2009). Polyamines as a common source of hydrogen peroxide in host- and nonhost hypersensitive response during pathogen infection. Plant Mol. Biol. 70, 103–112. 10.1007/s11103-009-9459-0, PMID: 19190986

[ref70] Zheng Y. Zhang Q. (2004). Effects of polyamines and salicylic acid on postharvest storage of “Ponkan” mandarin. Acta Hortic. 632, 317–320. 10.17660/ActaHortic.2004.632.41

